# Characterizing the convergence of protein kinase CK2 and caspase-3 reveals isoform-specific phosphorylation of caspase-3 by CK2α′: implications for pathological roles of CK2 in promoting cancer cell survival

**DOI:** 10.18632/oncotarget.948

**Published:** 2013-03-31

**Authors:** Jacob P. Turowec, Greg Vilk, Michelle Gabriel, David W. Litchfield

**Affiliations:** ^1^ Department of Biochemistry, Schulich School of Medicine, Western University, London, ON, Canada

**Keywords:** Protein kinase CK2, isozyme specific substrate, caspase-3

## Abstract

Protein kinase CK2 has emerged as a promising candidate for the treatment of a number of cancers. This enzyme is comprised of two catalytic subunits (CK2 and/or CK2α′) that form complexes with homodimers of regulatory CK2β subunits. While catalytic and regulatory CK2 subunits are generally expressed at similar levels to form tetrameric complexes, asymmetric expression of CK2 subunits has been associated with various forms of cancer and the enhanced survival of cancer cells. To elucidate mechanisms responsible for regulation of cancer cell survival by CK2, we recently employed computational and experimental strategies that revealed widespread overlap between sites for CK2 phosphorylation and caspase cleavage. Among candidates with overlapping CK2 and caspase cleavage sites was caspase-3 that is phosphorylated by CK2 to prevent its activation by upstream caspases. To elucidate the precise relationship between CK2 and caspase-3, we modulated expression of individual CK2 subunits and demonstrated that CK2α′ exhibits a striking preference for caspase-3 phosphorylation in cells as compared to CK2α and that CK2β exhibits the capacity to abolish caspase-3 phosphorylation. Since caspase-3 represents the first CK2 substrate selectively phosphorylated by CK2α′ in cells, our work highlights divergent functions of the different forms of CK2. Given the involvement of CK2 in a diverse series of biological events and its association with various cancers, this work has important implications for identifying pathological roles of distinct forms of CK2 that could instruct efforts to selectively target individual CK2 subunits for therapy.

## INTRODUCTION

Protein kinase CK2 is a ubiquitously expressed, highly conserved, constitutively active protein Ser/Thr kinase implicated in a plethora of cellular functions, including cancer progression [1]. The enzyme is generally comprised of two catalytic (CK2α or CK2α′) subunits that form a holoenzyme complex when bound with two regulatory CK2β subunits [2]. High levels of transcript expression [3, 4], as well as increased protein and kinase activity are associated with the pathological functions of CK2 in a number of human tumors and lymphomas (reviewed in [5]). Indeed, a causative role for CK2 in transformation was observed in mice where tissue-specific overexpression of CK2α in mammary and T-cells increased cancer burden [6, 7]. Interest in CK2 as a target for cancer therapeutics continues to mount with the number of patents filed for CK2 inhibitors increasing significantly in recent years [8] and at least one CK2 inhibitor, CX-4945, entering clinical trials [9]. Implicit in understanding how CK2 inhibition can be most successfully translated to the clinic will be a careful dissection of CK2 function in disease to distinguish between physiological roles that promote survival of healthy cells and pathological roles that enable the inappropriate growth and survival of diseased cells. Interestingly, recent studies have revealed mis-balanced expression of the CK2 subunits in breast cancer[10] and in head and neck cancer [11], although the precise mechanisms by which the unbalanced expression of CK2 subunits can impact disease progression remains largely unexplored.

The two catalytic subunits of CK2 are almost 90% identical within their catalytic domain, but exhibit divergent C-termini [12-15]. Not surprisingly, CK2α and CK2α′ display shared enzymatic characteristics including a minimal consensus phosphorylation site of S/T-X-X-D/E, suggesting that any isozyme-specific cellular functions/substrates are governed through subtle regulatory processes. In this respect, the unique C-terminus of CK2α has a number of mitotic phosphorylation sites that promote specific interactions with Pin-1 and the mitotic spindle [16, 17]. Despite its similarity to CK2α′, the catalytic domain of CK2α also appears to govern some isozyme-specific interactions including CKIP-1 [18], PP2A [19], and a tighter interaction with the regulatory CK2β subunit compared to CK2α′ [20]. Interestingly, while CK2β generally promotes phosphorylation of CK2 substrates, a small subset of targets – exemplified by calmodulin – are phosphorylated only in the absence of CK2β [21, 22]. Despite a divergence in binding affinities between CK2β and CK2α or CK2α′, the phosphorylation of substrates specific to the catalytic form of CK2 has not been systematically investigated in cells. Furthermore, the observation that CK2β modulates substrate specificity may be particularly important with respect to CK2-related disease pathology, as a high CK2α:CK2β ratio drives epithelial-to-mesenchymal transition in human breast cancer [10] and transgenic mice that over-express only CK2α in the mammary gland are more prone to adenocarcinomas than control litter mates [6].

The functional consequence of CK2 over-expression in human cancer remains poorly defined, likely due in part to the vast number (thousands) of substrates predicted to be phosphorylated by CK2 [23]. With respect to the cancer promoting functions of CK2, an anti-apoptotic role has been observed in a number of different studies (reviewed in [5]). While the majority of studies that involve CK2 in cancer are focused on CK2α, CK2α′^−/−^ mice display a hyper-apoptotic phenotype in spermatocytes [24] and forced expression of kinase-inactive CK2α′ in U2-OS cells leads to defects in growth [25]. Intriguingly, a number of CK2 substrates, including Bid [26] and YY1 [27], are also caspase substrates, and are phosphorylated proximal to their scissile bond where phosphorylation blocks both caspase cleavage, and the progression of apoptosis (reviewed in [28]). To systematically investigate the relationship between CK2 phosphorylation and caspase cleaveage, we performed computational and experimental studies employing peptide arrays that revealed a broad spectrum of proteins with overlapping CK2 and caspase recognition motifs [29]. One candidate with overlapping CK2 and caspase consensus motifs was caspase-3 which can be phosphorylated by CK2 at the residues directly flanking the scissile bond that directs its cleavage and caspase-3 activation. Notably, phosphorylation at these positions blocked caspase-3 cleavage by both caspase-8 and -9.

To elucidate the precise relationship between CK2 and caspase-3, we modulated expression of individual CK2 subunits and developed phospho-specific antibodies to monitor the phosphorylation of caspase-3 in cells. These studies revealed that CK2α′ exhibits a striking preference over CK2α for caspase-3 phosphorylation in cells. Interestingly, this preference is not observed with recombinant proteins *in vitro*. Furthermore, we demonstrated that CK2β negatively regulates caspase-3 phosphorylation in cells suggesting that the asymmetric expression of CK2 subunits can differentially affect caspase activation and cancer cell survival.

## RESULTS

### CK2α′ preferentially phosphorylates caspase-3

**Figure 1:**
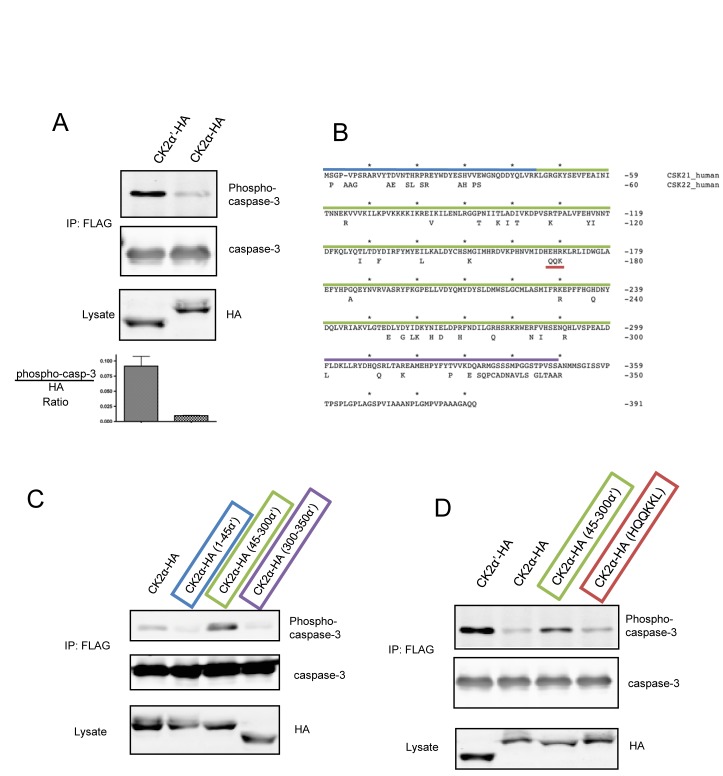
C3-FLAG is phosphorylated by CK2α′-HA, but not CK2α-HA in cells (A) HeLa-Tet-Off cells were transfected with C3-FLAG and CK2α-HA or CK2α′-HA, lysed, and C3-FLAG isolated by immunoprecipitation. Lysates and immunoprecipitations were blotted as indicated. Densitometric analysis represents the ratio of pC3:HA signal with error bars generated from the standard deviation of 3 independent experiments. (B) Sequence alignment of human CK2α (above) and α′ (lower). Residues that are not identical in CK2α′ are indicated and the segments used for chimeras are highlighted: residues 1-45 in blue, 45-300 in green, and 300-350 in purple and the motif responsible for PP2A binding by CK2α in red. (C and D) Cells were co-transfected with CK2 chimeras and C3-FLAG, lysed, and C3-FLAG enriched by immunoprecipitation. Lysates and immunoprecipitated samples were blotted as indicated.

We previously reported that caspase-3 is phosphorylated by recombinant CK2 at T174 and S176 – a phosphorylation event that inhibits activation by upstream caspases [29]. To further investigate caspase-3 phosphorylation, we raised antibodies against synthetic peptides of the doubly phosphorylated caspase-3 peptide (which are capable of detecting both singly and doubly phosphorylated and caspase-3 [30]) and systematically modulated expression of the CK2 catalytic isoforms to test phosphorylation of caspase-3-FLAG-(C163A). Interestingly, despite equal expression and activity of CK2α-HA and α′-HA, caspase-3 phosphorylation was preferentially observed in the presence of CK2α′-HA (Figure 1A, Supplemental Figure 1) [12-15]. To the best of our knowledge, this is the first demonstration that the cellular isozymes of CK2 can exhibit different activities against a substrate in cells – an observation especially remarkable given the 86% identity within their catalytic domain (residues 45-312) (Figure 1B). In an effort to prevent undesired caspase-3 activation, we utilized caspase-3-FLAG-(C163A) which replaces the catalytic cysteine with alanine. Interestingly, we found that caspase-3-FLAG was less efficiently phosphorylated than caspase-3-FLAG-(C163A) (hereafter referred to as C3-FLAG) (Supplemental Figure 1C).

To characterize sequence determinants responsible for isozymic preference of caspase-3 phosphorylation, we constructed CK2α / α′ chimeras in which three different segments of CK2α′ were swapped into CK2α-HA (Figure 1 C, upper panel). Segments consisted of the N-terminus (1-45), the catalytic domain that contains the most of the conserved subdomains for kinase family members (45-300), and the divergent C-terminus (300-350). Surprisingly, only CK2α-HA constructs containing amino acids 45-300 of CK2α′ showed phosphorylation of caspase-3 greater than CK2α-HA suggesting that the capacity to phosphorylate caspase-3 resides within the catalytic domain rather than less conserved regions of CK2α' (Figure 1C). Notably, though considerable divergence exists between the C-termini of CK2α and CK2α′, this domain does not play a role in dictating specificity towards caspase-3. Interestingly, amongst the primary sequence differences between the catalytic domain of CK2α and CK2α′ is the conspicuous presence of a highly conserved HEHRKL motif in CK2α that is absent in CK2α′ and previously reported to be required for binding PP2A [19] (Figure 1B). Several investigators have hypothesized that this region could serve as a distinguishing feature for PP2A interaction, and isoform specific functions of catalytic CK2 [31, 32]. Therefore, we hypothesized that a PP2A/CK2α-HA interaction could reduce C3-FLAG phosphorylation, and so aimed to test this by swapping the CK2α′ HQQKKL sequence into CK2α-HA and probing for C3-FLAG phosphorylation. However, CK2α-HA(HQQKKL) was unable to rescue C3-FLAG phosphorylation, arguing against a PP2A-CK2α complex that dictates isoform specific phosphorylation of caspase-3 (Figure 1D).

### Phosphorylation of caspase-3 by CK2a' is attenuated by CK2β

**Figure 2:**
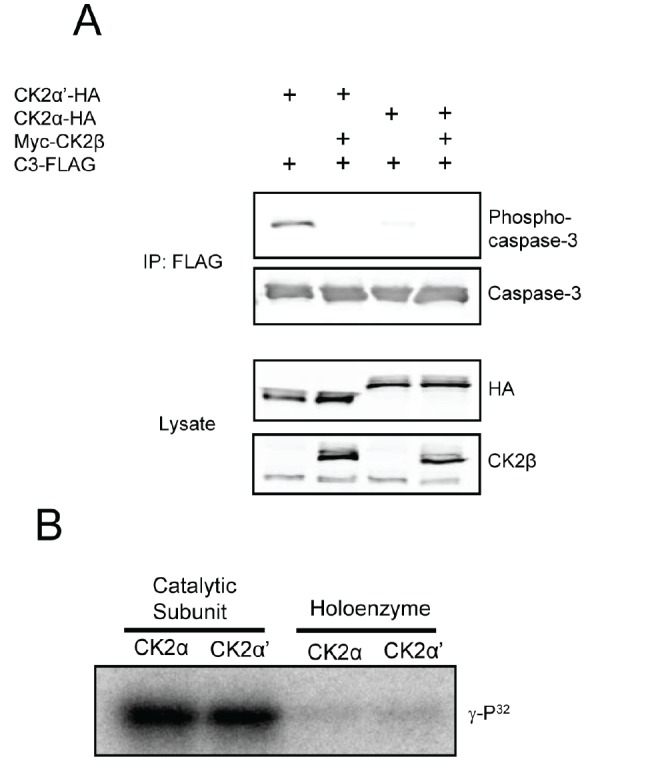
CK2β inhibits caspase-3 phosphorylation (A) Cells were co-transfected with the indicated CK2 constructs and C3-FLAG. Lysates were immunoprecipitated with anti-FLAG to isolate caspase-3, separated by SDS-PAGE and immunoblotted as indicated. (B) Equal units of the indicated forms of recombinant CK2 were used in kinase reactions with caspase-3-His (C163A) and ATP-γ-P^32^. Reactions were separated by SDS-PAGE, the gels dried, and visualized using a phosphorimager.

The regulatory CK2β subunit is capable of modulating substrate specificity of CK2 despite having no known catalytic activity. Most substrates are phosphorylated more readily by the CK2 holoenzyme *in vitro* – a characteristic likely promoted by the enhanced stability of CK2α or CK2α′ in complex with CK2β – although a small subset of substrates, exemplified by calmodulin, are phosphorylated only in the absence of CK2β [21, 22]. Interestingly, CK2α′ binds CK2β more than 10× weaker *in vitro* than does CK2α as a result of altered folding of the β4/β5 loop – a structural feature within the catalytic domain that makes significant contacts with CK2β [20]. Therefore, we were driven to first test the hypothesis that CK2β regulates phosphorylation of caspase-3, and second, to investigate whether the differential affinity for CK2β exhibited by the two catalytic subunits might impart isozymic substrate preferences. Upon co-transfection of myc-CK2β with either catalytic isozyme and C3-FLAG, we found that CK2β dramatically attenuated C3-FLAG phosphorylation by CK2α′-HA and further reduced the low level of phosphorylation achieved by CK2α-HA expression (Figure 2A). CK2β also blocked phosphorylation of C3-FLAG by myc-CK2α′ in U2-OS cells (data not shown). The ability of CK2β to block caspase-3 phosphorylation in cells prompted us to test all forms of CK2 for their ability to phosphorylate caspase-3 in kinase assays using recombinant proteins. Figure 2B shows that isozymic specificity was lost when *in vitro* kinase assays were performed, but the inhibitory effect of CK2β remained. Furthermore, like other CK2 substrates that are phosphorylated only in the absence of CK2β, treatment of the holoenzyme with polyamines resulted in hyperphosphorylation of caspase-3 in *in vitro* kinase assays (data not shown).

### CK2 is predominantly within holoenzyme complexes in cells

**Figure 3:**
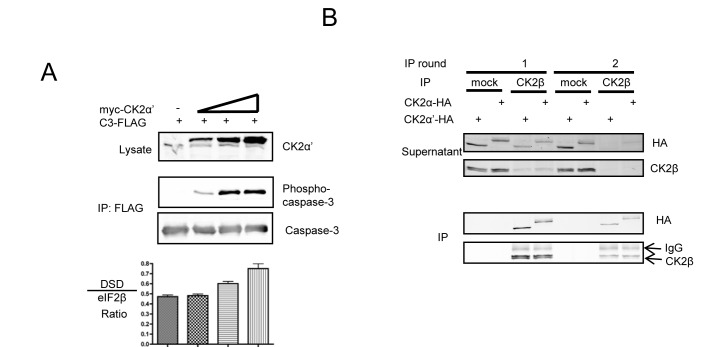
Investigation of CK2 form in cell lysates reveals a predominately holoenzyme form (A) Cells were transfected with C3-FLAG and varying amounts of myc-CK2α′, and harvested after 40 h. Lysates and FLAG immunoprecipations were probed as indicated. Lysates were assessed in kinase assays along with two CK2 substrate peptides. The DSD peptide (RRRDDDSDDD) is phosphorylated by both catalytic free CK2 and holoenzyme while the eIF2β peptide (MSGDEMIFDPTMSKKKKKKKKP) is phosphorylated exclusively by the CK2 holoenzyme. Graphs represent the ratio of DSD:eiF2β activity with error bars representing the standard deviation of triplicate kinase assays. (B) Cells transfected with CK2α-HA or CK2α′-HA were harvested and subjected to consecutive rounds of immunoprecipitation using a CK2β antibody or mock control. Both the supernatant from the immunoprecipitation and the immunoprecipitation itself were western blotted for HA and CK2β as indicated.

In an effort to investigate if significant pools of CK2α′ devoid of CK2β were present after over-expression, we utilized a CK2 substrate peptide that does not distinguish between catalytic subunits or the holoenzyme (DSD in Figure 3A) and an eIF2β substrate peptide that is specific for the holoenzyme [33]. An increase in the DSD:eIF2β ratio indeed suggested an increase in CK2β-free myc-CK2α′ (Figure 3A). Of particular interest was the observation that C3-FLAG phosphorylation was actually detected before measurable differences in DSD:eIF2β phosphorylation, suggesting that the in vitro assay either lacks the required sensitivity to detect small changes in the ratio of free catalytic subunits and holoenzyme or that a cellular, CK2β-refractory population of CK2α′ becomes complexed with CK2β upon cell lysis. In support of the latter, we also observed complete complex formation between HA-tagged CK2 catalytic subunits with endogenous CK2β (Figure 3B). Here, lysates subjected to immunoprecipitation with CK2β antibodies show over 90% depletion of CK2β, CK2α-HA and CK2α′-HA after two rounds of immunoprecipitation, with no substantial difference between the catalytic subunits remaining in the supernatant. That HA-tagged CK2α and CK2α′ bound endogenous CK2β further reinforces the notion that both isozymes of ectopic CK2 are fully functional, and that the difference in caspase-3 phosphorylation may arise from differences in the cellular regulation of CK2α-HA versus CK2α′-HA that extend exclusively beyond regulation by CK2β. Furthermore, in spite of the previously demonstration that CK2α′ binds less efficiently to CK2β as compared to CK2α [20], it did not appear that there was an appreciable difference in holoenzyme formation between the two catalytic subunits in cell lysates.

When examining the phosphorylation of caspase-3 in HeLa cells, we found that endogenous CK2 was unable to appreciably phosphorylate C3-FLAG suggesting that in HeLa cells, CK2α′ is exclusively in holoenzyme complexes with CK2β (Figure 3A, Lane 1). Under some circumstances, CK2 holoenzyme-mediated phosphorylation can be stimulated by polyamines in *in vitro* kinase assays and by over-expression of ODC, the rate limiting enzyme of spermine synthesis, in cells [34-36]. However, in the case of caspase-3, over-expression of ODC1 did not promote its phosphorylation (data not shown).

### Nuclear redistribution of caspase-3 does not promote phosphorylation by CK2α

**Figure 4:**
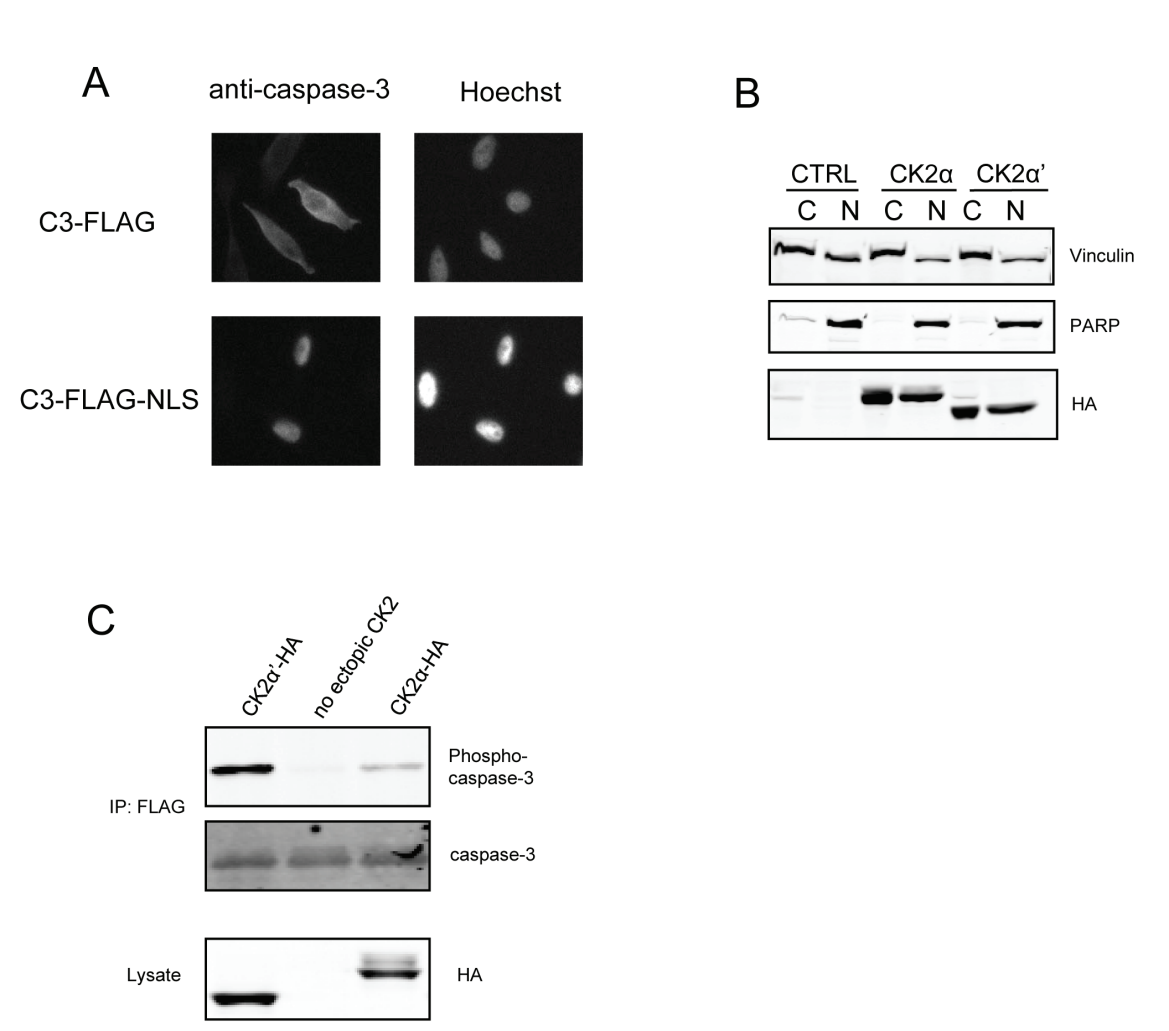
Redirecting C3-FLAG to the nucleus does not alter the relative levels of phosphorylation (A) Cells were transfected with C3-FLAG or C3-FLAG-NLS and then transferred to glass cover slips in 6-well dishes. Twenty-four hours after transfection, cover slips were stained using caspase-3, which was detected by Alexa-Fluor GAM-488, and Hoechst 33258, mounted on microscope slides and analyzed on an inverted fluorescence microscope. (B) Cells were transfected with CK2α-HA or CK2α′-HA and the cytoplasmic and nuclear fractions separated and blotted as indicated. PARP was used as a marker for nuclear separation and Vinculin for cytoplasmic. (C) Cells were co-transfected with C3-FLAG-NLS and the indicated CK2 construct, then harvested. FLAG immunoprecipitations and lysates were immunoblotted as indicated. CTRL denotes the empty vector lane.

A striking finding from studies described above (Figure 1) is the preferential phosphorylation of caspase-3 by CK2α′ as compared to CK2α. Since these two CK2 isoforms exhibit very similar enzymatic characteristics in terms of substrate specificity, we undertook an investigation of other functional features that might contribute to the selective phosphorylation of caspase-3 by CK2α′. While both isoforms of CK2 have been identified within several cellular compartments, including the nucleus and cytoplasm, we were particularly intrigued by the observations of Filhol and colleagues who demonstrated that GFP-CK2α localizes to the nucleus in the absence of CK2β [37]. A prediction from their observations is that CK2 is predominantly within tetrameric holoenzyme complexes within the cytoplasm where caspase-3 is predominantly localized, whereas some free CK2α may reside within the nucleus. Consequently, we were interested in determining whether caspase-3 would exhibit enhanced phosphorylation if it were localized to the nucleus. By fusing a nuclear localization signal (NLS) on the C-terminus of C3-FLAG, caspase-3 was redirected to the nucleus (Figure 4A, lower panels). However, even when it was localized to the nucleus (Figure 4C), caspase-3 was preferentially phosphorylated by CK2α′. Overall, these data suggest that the preferential phosphorylation of caspase-3 by CK2α' is not simply a consequence of its nuclear versus cytoplasmic localization.

### The isozymic preference of caspase-3 phosphorylation is abolished by okadaic acid

**Figure 5:**
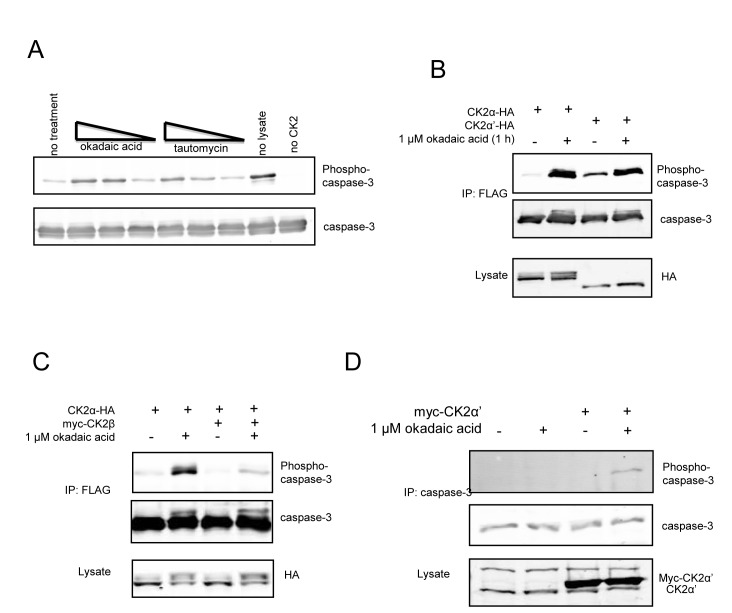
Exclusion of CK2α-HA from C3-FLAG phosphorylation is not governed by endogenous CK2β in HeLa cells (A) Recombinant His-tagged caspase-3-(C163A) was fully phosphorylated by GST-CK2α. Reactions were quenched by the addition of the CK2 specific inhibitor TBBz, and added to HeLa lysates harvested in the absence of phosphatase inhibitors. Okadaic acid or tautomycin at concentrations of 50, 5 or 0.5 nM was added to the reaction as indicated. (B-D) HeLa cells were transfected with C3-FLAG and the indicated constructs except in (D), where endogenous caspase-3 was being probed. After a 24 hour recovery, cells were treated with or without 1 uM okadaic. FLAG or caspase-3 immunoprecipitations and lysates were blotted as indicated.

The inability of CK2α to promote phosphorylation of caspase-3 in cells suggested either that CK2α does not phosphorylate caspase-3 in cells or that phosphorylation is readily reversed by dephosphorylation. Indeed, when recombinant caspase-3 is phosphorylated, and then incubated with cellular extracts lacking phosphatase inhibitors, a marked decrease in phosphorylation was observed indicating active dephosphorylation (Figure 5A). Furthermore, dephosphorylation was more sensitive to okadaic acid (Ki for PP2A < PP1) than tautomycin (Ki for PP2A > PP1), suggesting PP2A as the responsible phosphatase. In cells, okadaic acid did not promote C3-FLAG phosphorylation in the absence of expression of free CK2 catalytic subunits, further suggesting that endogenous CK2α′ is fully bound to CK2β (data not shown). By comparison, okadaic acid completely abolished the isozyme preference for caspase-3 phosphorylation. In this respect, okadaic acid promoted phosphorylation of caspase-3 by CK2α-HA to levels comparable to those seen with CK2α′-HA (Figure 5B). As was the case in the absence of okadaic acid, phosphorylation was blocked by myc-CK2β (Figure 5C). One possible explanation for these observations is that there is sufficient CK2α-HA that is free of CK2β to be catalytically competent to phosphorylate C3-FLAG, but its ability to phosphorylate caspase-3 is restricted by a mechanism that is relieved by okadaic acid. Notably, we also observed that endogenous caspase-3 was phosphorylated by ectopic CK2α′ in the presence of okadaic acid (Figure 5D). Overall, our results support the conclusion that caspase-3 phosphorylation could be physiologically relevant in tissues or pathologies where there are free CK2 catalytic subunits, most notably CK2α′ that displays a striking preference over CK2α for caspase-3 phosphorylation.

## DISCUSSION

On the basis of its aberrant expression or activity in a number of human cancers, protein kinase CK2 has emerged as an attractive candidate for molecular-targeted therapy. While CK2 is generally considered to be a tetrameric enzyme comprised of balanced levels of catalytic and regulatory subunits, asymmetric expression of CK2 subunits has been observed in a number of tumors. Perhaps the first indication of a CK2β-free form of catalytic CK2 in cells was the identification of phosphorylation sites on calmodulin from rat liver that could only be phosphorylated by catalytic CK2 subunits *in vitro* [38]. A more direct indication for the asymmetric expression of CK2 subunits comes from the studies of Deshiere *et al.* (2012) who noted that the CK2α′:CK2α:CK2β ratio varies across breast cancer samples, and that a higher CK2α:CK2β ratio correlates with the expression of epithelial-to-mesenchymal transition markers [10]. Collectively, these studies provide evidence for the existence of free catalyic CK2 subunits in cells. However, a comprehensive understanding of the precise roles of individual CK2 forms in physiology and in disease remains to be established.

By manipulating the expression of individual forms of CK2 to investigate the relationship between CK2 and caspase pathways [29], we have observed a striking preference of CK2α′ for the phosphorylation of caspase-3 in cells. In this respect, phosphorylation of caspase-3 by CK2α′ is abolished by CK2β and CK2α, either in the presence or absence of CK2β exhibits a dramatically reduced capacity to phosphorylate caspase-3 as compared to CK2α′. Prior to this study, some CK2α′ specific functions had been observed, the most notable of which is the finding that CK2α′ knock-out mice exhibit a hyper-apoptotic phenotype in spermatocytes [24], and that forced expression of kinase-inactive CK2α′ in U2-OS cells resulted in proliferation defects [25]. Furthermore, Orlandini *et al.* (1999) highlighted the oncogenic activity of CK2α′ in experiments that demonstrated co-operativity with Ras in the transformation of rat fibroblasts [39]. In a related vein, Li *et al.* (2006) showed that NKX3.1 has a decreased half-life upon CK2α′ knock-down, or mutation of phospho-acceptors to alanine, and that NKX3.1 phosphorylation was mediated by CK2α′ devoid of CK2β in in-gel kinase assays [40]. By identifying a CK2 substrate preferentially phosphorylated by CK2α′, our studies reinforce the notion that CK2α′ has cellular functions distinct from CK2α and, in doing so, develop a biomarker that might aid in identifying cells with functional levels of free CK2α′.

To elucidate the molecular basis for the preferential phosphorylation of caspase-3 by CK2α′, we generated chimeras that enabled CK2α-mediated phosphorylation of caspase-3 when constructs contained residues 45-300 within the catalytic domain of CK2α. What is striking is that a comparison of the CK2α and CK2α′ sequences reveals this internal catalytic portion as actually the most conserved – residues 1-45 are 74% identical, 45-300 are 88% identical and 300-350 are 56% identical. Interestingly, the catalytic domain of CK2α has previously been implicated in isoform specific interactions. For example, the Goldberg laboratory demonstrated that PP2A interacts with the HEHRKL motif within CK2α – an area with a cluster of divergence from CK2α′ (Figure 1B) – though we found that swapping the HQQKKL motif of CK2α′ into CK2α was unable to promote C3-FLAG phosphorylation [19]. Similarly, this region has also been tested for regulating the CK2 specific binding to CKIP-1, but CK2α-E167Q was unable to disrupt CKIP-1 interaction, nor was CK2′-Q168E able to promote binding to CKIP-1 [41]. The observation that residues 45-300 of CK2α′ mediate C3-FLAG phosphorylation further reinforces the notion that subtle regulatory mechanisms must exist to differentially control the two CK2 isozymes.

Structures of each of the CK2 catalytic subunits determined by X-ray crystallography do reveal some structural variation within residues 45-300 of the catalytic subunits although differences also involve contributions from the N-terminus. For example, the β4/β5 loop is constitutively open in CK2α′ and is located at residues 95-114, but stabilization is in part mediated by residues 34-37 and thought to result from a unique proline at residue 32 in CK2α′ [20]. Interestingly, Pro^92^ is unique to CK2α and located just upstream of the β4/β5 loop, though the changes that result from this divergence remain unknown. Another structural variation occurs in the interdomain region – a segment responsible for co-ordinating binding with ATP and ATP-competitive inhibitors (residues 114-122 of CK2α′). This hinge is slightly collapsed in CK2α, but also correlates with a closed β4/β5 loop which, again, appears to receive structural contributions from the N-terminus. Tyr^116^ and Ile^117^ are not conserved in CK2α (His^115^, Val^116^) but how these residues contribute to differences in hinge region structure remain unknown. Should the noted structural alterations be consistent with our chimeras, it is intriguing to speculate that okadaic acid either governs protein-protein interactions or post-translational modifications of CK2α that can resolve this divergence. With respect to the latter, a search of the PHOSIDA database to identify unique post-translational modifications on either CK2α or CK2α′ revealed that CK2α′ is acetylated on K97 (T96 on CK2α) and CK2α phosphorylated on T127 (I128 on CK2α′), providing a putative mechanism that differentially controls CK2 catalytic isozymes [42]. Precedence for the former has been observed with v-src as okadaic acid treatment disrupts the v-src/Hsp90 interaction [43]. Interestingly, CK2α also interacts with Hsp90 [44, 45], but whether this interaction restricts access to CK2 substrates or is altered by okadaic acid remains speculative.

Studies with recombinant proteins have revealed that CK2β binds to CK2α and CK2α′ spontaneously in solution [46] with a low Kd (CK2α′ − 45.5 × 10^−9^ M, CK2α − 3.7 × 10^−9^ M [20]). Furthermore, dissociation of the catalytic and regulatory CK2 subunits has only be observed under denaturing conditions [47]. By comparison, more recent studies performed by expressing fluorescent variants of CK2 subunits in cells clearly suggest that there is a dynamic assembly and disassembly of CK2 complexes within cells [37]. Consequently while immunoprecipitation assays suggest that CK2α and CK2α′ are predominantly within CK2β-containing complexes that would be unable to phosphorylate caspase-3, it is likely that the immunoprecipiation assays do not accurately reveal the dynamics of CK2 complexes in cells. Furthermore, when considering that CK2β has been shown both in cell lines and in tissues to be present in excess of the CK2 catalytic subunits, [48, 49], it is not inconceivable that CK2 complexes form following cell lysis. Taken together with the demonstration that CK2 complexes undergo a dynamic assembly and disassembly in cells, our demonstration that caspase-3 is phosphorylated by CK2α in cells provides compelling support for the existence of free catalytic CK2 subunits in cells. Furthermore, based on our findings, it would appear that the phosphorylation of caspase-3 may represent a biomarker for identifying cells with free CK2α′.

Based on our recent demonstration of an extensive repertoire of proteins with overlapping consensus recognition motifs for CK2 and for caspases, it is also important to emphasize that cells that express aberrant levels of CK2 may exhibit alterations in caspase actions. In this respect, we identified numerous proteins that are candidate substrates for both CK2 and caspases, where the CK2 phosphorylation site is positioned at an inhibitory position within the caspase motif and may function to attenuate caspase action [29]. Given that CK2 is elevated in numerous cancers and is constitutively active, our earlier work raises the prospect that the elevated levels of CK2 that accompany malignancies leads to enhanced survival through the phosphorylation of pathological substrates that prevent their cleavage by caspases [29]. One of the pathological substrates for CK2 that we identified in our study was caspase-3. Notably, we demonstrated that phosphorylation of caspase-3 by CK2 could abrogate its cleavage by caspases 8 or 9. In the present work, we have further examined this model by investigating the phosphorylation of caspase-3 by different forms of CK2 in cells. Since caspase-3 represents the first CK2 substrate selectively phosphorylated by CK2α′ in cells, our work highlights divergent functions of the different forms of CK2. In this respect, although speculative at this stage, cancers that exhibit elevated levels of CK2α′, especially in the absence of CK2β, that are accompanied by pathological levels of caspase-3 phosphorylation may be candidates for CK2 inhibition. More generally, we anticipate that the phosphorylation status of other pathological CK2 substrates will also be dictated by the active complement of free-catalytic or holoenzyme present. With the emergence of CK2 as a candidate for molecular-targeted therapy, including the introduction of CK2 inhibitors in clinical trials, a thorough understanding of the roles of CK2 both in normal physiology and in disease offers the promise of guiding the development of CK2-targeted interventions.

## MATERIALS AND METHODS

### Cell culture and transfections

HeLa (tet-Off) cells (Clontech) were cultured in Dulbecco's modified Eagle's medium (DMEM) containing 10% FBS, penicillin (100 U/mL), and streptomycin (100 mg/mL) on 10-cm dishes. Cells were transfected using 40 μg of total plasmid DNA via the calcium phosphate method [50]. For phosphatase inhibition studies, cells were treated with okadaic acid (Bioshop).

### Plasmid Construction

CK2α-HA, CK2α′-HA, caspase-3-FLAG (C163A), caspase-3-his (C163A) and myc-CK2α were prepared as described elsewhere [29, 51]. CK2α-HA (1-45 CK2α′) and CK2α-HA (300-350 CK2α′) were constructed by first subcloning CK2α-HA from pRC/CMV into pet28a using HindIII and ApaI. CK2′-HA was cut with BstBI / Bsu36I, to obtain the 1-45 amino acid fragment of CK2α′, and ligated into the pet28a-CK2-HA. This subclone was then moved back to pRC/CMV using HindIII / XbaI. For 300-350 CK2α′, CK2α′-HA was ligated into pRC/CMV-CK2α-HA after digestion with Bsu36I and ApaI. 45-300 CK2α′ was cloned using overlapping DNA sub-fragments generated by PCR reactions with T7 (F 5′-TAA TAC GAC TCA CTA TAG GG-3′) and reverse 5′-CCC CGA CCA AGT TTT CGA ACC AGC TGG TAG TCA TC-3′ (for 1-45 CK2α), forward 5′-GAT GAC TAC CAG CTG GTT CGA AAA CTT GGT CGG GG-3′ and reverse 5′-GTC CAG GAA ATC CAA GGC CTC AGG GCT GAC AAG GTG-3′ (for 45-300 CK2α′) and forward 5′-CAC CTT GTC AGC CCT GAG GCC TTG GAT TTC CTG GAC-3′ and Sp6 (R 5-CAG CGA GCT CTA GCA TTT AGG TGA CAC TAT AG-3′) (for 300-391 CK2α-HA). Sub-fragments were gel purified, mixed, PCR amplified for 2 cycles, amplified for another 20 cycles with T7 and Sp6, ligated into pCR-BLUNT, and cloned back into pRC/CMV using HindIII and ApaI. Caspase-3-FLAG-NLS-(C163A) was generated by performing PCR reactions on caspase-3-FLAG-(C163A) with T7 (F) and a reverse primer that included an NLS tag (PKKKRKV) and a XhoI restriction site downstream of the FLAG tag (5′-TTC TCG AGC TAC ACC TTA CGC TTT TTC TTT GGC TTG TCG TCG TCG TCC TT-3′). The PCR product was ligated into PCR-Blunt, digested out using BamHI and XhoI and ligated into pCDNA3.1(+). All constructs were sequence verified.

### Cell lysis, immuno-precipitations and Western blotting

Cells were lysed in NP-40 alternative lysis buffer [1% NP-40 alternative, 150 mM NaCl, 50 mM Tris (pH 7.5), leupeptin (10 μg/mL), 0.1 mM phenylmethylsulfonyl fluoride (PMSF), pepstatin (10 μg/mL), aprotinin (5 μg/mL), 25 mM NaF, 1 μM okadaic acid and 1 mM sodium orthovanadate]. Lysates were sonicated 2 × 5 s, spun for 15 minutes at 4°C at 13 000 × *g*, and protein concentrations determined by bicinchoninic acid (BCA) assay (Thermo Scientific). For immuno-precipitations, 0.5 – 2 mg of lysate was tumbled 4 μg of anti-Flag M2 (Sigma) and 30 μL of protein A/G sepharose beads (GE Healthcare) for 1 H. For CK2β immuno-precipitations, anti-CK2β sera [2] was tumbled with 200 ug of lysate for 1 H, then 30 μL of protein A sepharose beads added and tumbled for an additional hour. Endogenous caspase-3 was immuno-precipitated from 10 mg of lysate with 5 μg of anti-caspase-3 (BD Biosciences clone 19) and 30 μL of protein A/G sepharose beads. Immune-complexes were washed 2 × 500 μL NP-40 alternative lysis buffer and proteins separated by 12% SDS-PAGE, transferred to PVDF membranes and blotted using antibodies to: caspase-3 (BD biosciences clone 19), HA (3F10, Roche), PARP (Cell Signaling Technology), Vinculin (Sigma), phospho-caspase-3 (pT174/pS176) [30], CK2 [52], CK2α′ [52], CK2β [52], SAV-680, GAM-680 and GAR-800 (LI-COR Biosciences). Quantitation of western blots was performed using Odyssey software (version 3.0) from LI-COR Biosciences.

### Nuclear and Cytoplasmic fractionation of transfected HeLa cells

CK2-HA was assessed in different cellular departments using the NE-PER Nuclear-Cytoplasmic extraction kit (Thermo Scientific). Briefly, transfected HeLa cells were lysed and subsequently treated according to manufacturer's recommendations in order to obtain purified whole cell extracts of nuclear or cytoplasmic origin. Using a BCA protein assay kit (Thermo Scientific), 40 or 80 μg of whole cell extract was loaded onto 10 or 15 % SDS-PAGE gels and the resolved proteins were transferred to PVDF membrane for subsequent Western Blotting using monoclonal and polyclonal antibodies targeted against caspase-3, phosphorylated caspase-3, CK2α, CK2α', and CK2β. Vinculin and PARP antibodies were used to monitor for purity of each cellular compartment.

### Kinase assays

Kinase assays using recombinant proteins were performed largely as described in [29]. Caspase-3-his-(C163A) and catalytic subunits of GST-tagged CK2 were purified from bacterial sources as previously noted in [29] and [53], respectively. Holoenzyme preparations of CK2 were prepared by expressing catalytic and regulatory subunits of CK2 in separate preparations of *E. coli*, mixing the pellets during lysis, and isolating the holoenzyme by purification on a heparin column and then by size exclusion chromatography. The activity of purified CK2 was characterized [53] by performing kinase assays with the substrate peptide RRRDDDSDDD (EZBiolab) (100 μM) so that equal units of enzyme could be added to kinase assays containing 1 μg of recombinant caspase-3-his-(C163A). All assays were performed in 50 mM Tris (pH 7.5), 150 mM NaCl, 10 mM MgCl_2_, 100 μM ATP and 0.2 μCi [γ-^32^P]ATP per 30 μL of reaction.

When using cell extracts as the kinase source, lysates were prepared by scraping cells into ice-cold 50 mM β-glycerophosphate, 1.5 mM EGTA, leupeptin (10 μg/mL), 0.1 mM phenylmethylsulfonyl fluoride (PMSF), pepstatin (10 μg/mL), aprotinin (5 μg/mL), 1 μM okadaic acid and 1 mM sodium orthovanadate, followed by sonicating for 2 × 5 s and removing debris by centrifuging for 15 minutes. After a Bradford assay, kinase assays were performed using 5 g of lysate, 100 μM RRRDDDSDDD or 100 μM of eIF2β peptide (MSGDEMIFDPTMSKKKKKKKKP) in 50 mM Tris (pH 7.5), 150 mM NaCl, 10 mM MgCl_2_, 100 μM ATP and 0.2 μCi [γ-^32^P] ATP per 30 μL of reaction. Assays were performed for 5 or 10 minutes, to verify linearity, and stopped by spotting on P81 paper. ^32^P was detected using a STORM phosphorimager and quantitated with ImageQuant TL software.

### Lysate dephosphorylation assay

Phospho-caspase-3 was generated by phosphorylating caspase-3-his (C163A) with excess GST-CK2α in 150 mM NaCl, 50 mM Tris (pH 7.5), 10 mM MgCl_2_, and 100 μM ATP. Reactions were diluted 1/50 into 100 μg of lysate such that 1 μg of phospho-caspase-3 was in each reaction. Fifty μM of the CK2 inhibitor TBBz (calbiochem) was included to stop the kinase assay. 500, 50 or 5 nM of okadaic acid (Bioshop) or tautomycin (calbiochem) was also added to the lysate dephosphorylation assay. DMSO was used as a carrier control.

### Microscopy

Cells were grown on sterile glass cover slips, washed with PBS, fixed with 3.7% paraformaldehyde for 30 minutes, washed in 0.1 M glycine for 20 minutes at 37°C and permeabilized with 0.1% Triton X-100 for 5 minutes. All solutions were made in PBS and incubations were performed at room temperature unless otherwise stated. After permeabilization, cells were incubated for 45 minutes with a 1:50 dilution of anti-caspase-3 in 5% BSA/PBS, washed 3× PBS and then incubated with GAM-AlexaFluor 488 (1:1000) (Invitrogen) for 45 minutes. Samples were stained with Hoechst 33258 before final PBS washes and mounting with ProLong Gold (Invitrogen). Slides were visualized using an Axiovert inverted fluorescence microscope.

## Supplementary Figures


