# Supplementary material for: Characterizing the convergence of protein kinase CK2 and caspase-3 reveals isoform-specific phosphorylation of caspase-3 by CK2α′: implications for pathological roles of CK2 in promoting cancer cell survival
Source: Oncotarget. 2013 Mar 31;4(4):560–71. doi: 10.18632/oncotarget.948 (PMC3720604; doi:10.18632/oncotarget.948)
Supplement: Supplementary file 1 [file oncotarget-04-560-s001.pdf]

Characterizing the convergence of protein kinase CK2 and caspase-3 reveals isoform-specific phosphorylation of caspase-3 by CK2α′: implications for pathological roles of CK2 in promoting cancer cell survival - Turowec et al

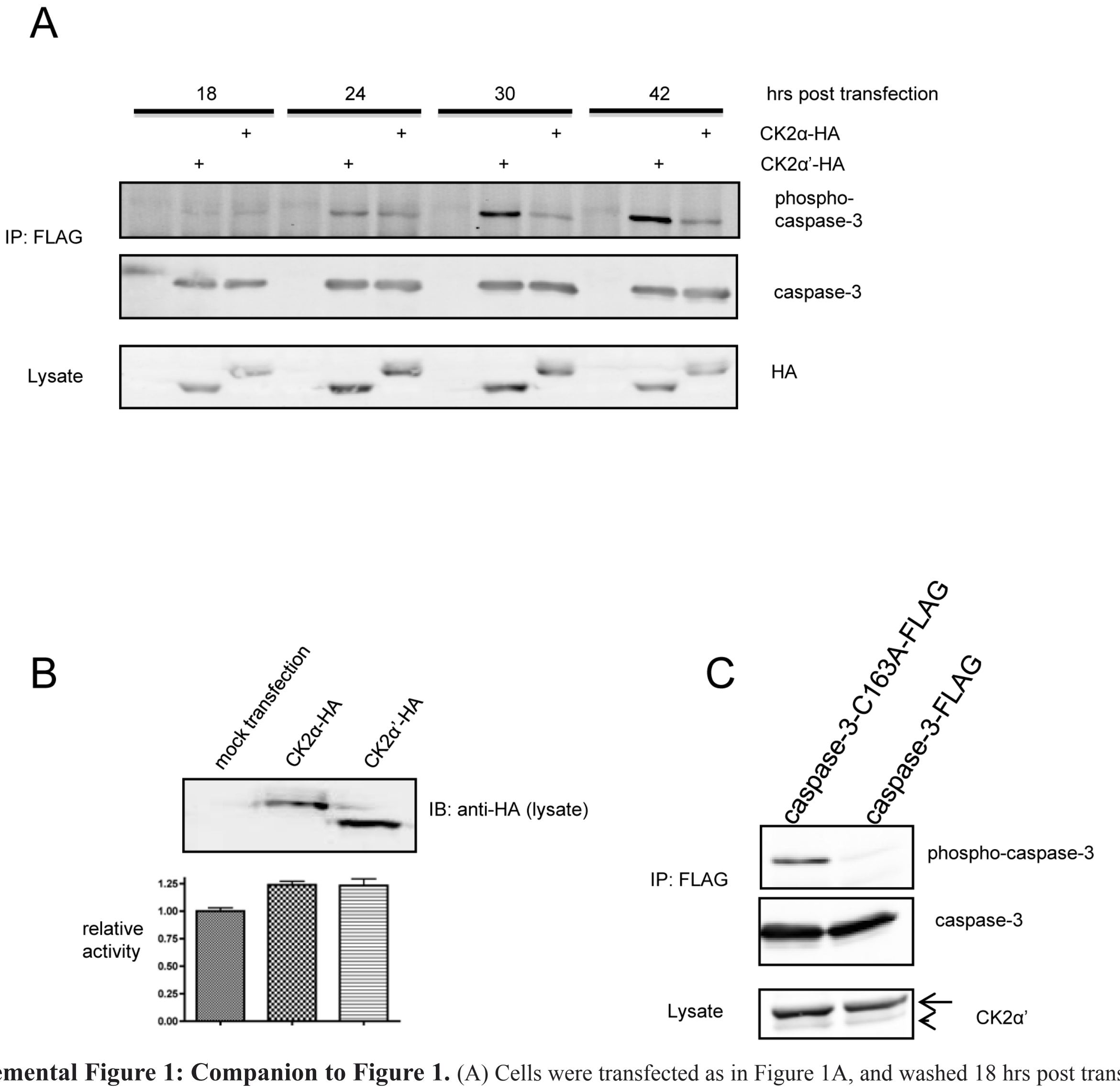

Supplemental Figure 1: Companion to Figure 1. (A) Cells were transfected as in Figure 1A, and washed 18 hrs post transfection. Lysates were generated at the indicated timepoints followed by FLAG immunoprecipitation and western blotting as indicated. (B) Lysates from HeLa cells exhibiting equal amounts of CK2α-HA and CK2α′-HA were used for kinase assays with the CK2 specific substrate peptide RRRDDDSDDD. Error bars represent the standard deviation of triplicate reactions. (C) Cells were transfected with myc-CK2α′ and the indicated caspase-3 construct, washed after 18 hrs and allowed to recover for 24 hrs. At this point lysates were generated, FLAG immunoprecipitations performed and samples western blotted as indicated.
